# Gut microbiota and atopic dermatitis in children: a scoping review

**DOI:** 10.1186/s12887-022-03390-3

**Published:** 2022-06-02

**Authors:** Yue Liu, Xiaofan Du, Shujie Zhai, Xiaodong Tang, Cuiling Liu, Weihong Li

**Affiliations:** 1 Department of Acupuncture and Tuina Science, School of Traditional Chinese Medicine, Naval Medical University, No. 800 Xiangyin Road, Shanghai, 200433 China; 2 Clinical Medicine Science, Anhui Medical University, Hefei, 230032 China; 3 Logistics Service Center, People’s Hospital of Xinjiang Uygur Autonomous Region, Urumqi, 830001 China

**Keywords:** Gut microbiota, Atopic dermatitis, Children, Probiotics, Review, Treatment, Prevention

## Abstract

**Background:**

Gut microbiota plays an important role in the development of atopic dermatitis (AD). We aimed to elucidate research trends in gut microbiota and AD in children, to provide evidence and insights to the clinical prevention and treatment of AD in children.

**Methods:**

A scoping literature review on the studies of gut microbiota and AD were conducted. Two authors independently searched Pubmed et al. databases for studies focused on gut microbiota and AD in children up to January 15, 2022. The literatures were screened and analyzed by two reviewers.

**Results:**

A total of 44 reports were finally included and analyzed. Current researches have indicated that abnormal human microecology is closely associated with AD, and the disturbance of intestinal microbiota plays an important role in the occurrence and development of AD. Probiotics can correct the microbiota disorder, have the functions of regulating immunity, antioxidant, and help to restore the microecological homeostasis. However, there is still a lack of high-quality research reports on the efficacy and safety of probiotics in the prevention and treatment of AD in children.

**Conclusions:**

The changes of gut microbiota are essential to the development of AD in children, which may be an effective target for the prevention and treatment of AD. Future studies with larger sample size and rigorous design are needed to elucidate the effects and safety of probiotics in AD.

## Introduction

Atopic dermatitis (AD) is a chronic, recurring, itchy skin disease with an incidence of 0.2% to 25% [1, 2]. Recent epidemiological survey data in China [3–6] have showed that the incidence of AD in children aged 3–6 years old can be as high as 18.3%, and it is increasing year by year. AD is more common in infants and young children, and AD usually reappears in adulthood period of AD children. Besides, AD children over 2 years old may have a longer course of disease and are difficult to cure. If the patient suffers from the disease for a long time and develops to the age of 20 to 30 years, the patient may even develop cataracts associated with AD [7]. Therefore, active and effective treatment and prevention of AD in children are of great significance to their growth and quality of life.

AD is a common chronic and relapsing skin disease in children with complex pathogenesis, which is related to multiple factors such as heredity, immune dysfunction, skin barrier damage, and environmental exposure [8, 9]. In recent years, with the development of high-throughput technology, the relationship between the human micro-ecosystem and AD has been gradually revealed. Some studies [10, 11] speculate that intestinal microbiota disturbance may be related to the pathogenesis and severity of AD. Previous studies [12, 13] have pointed out that the composition of the gut microbiota is significantly different between healthy children and children with allergic diseases. Changes of gut microbiota is associated with immune dysfunction in children [14, 15]. However, there are relatively few studies on gut microbiota and AD in children, and the current research status is unclear. Therefore, this scoping review aimed to review the studies on the gut microbiota and AD in children, to provide evidence for the clinical prevention and treatment of AD.

## Methods

This scoping review was conducted according to the Preferred Reporting Items for Systematic Reviews and Meta-analysis(PRISMA) extension for Scoping Reviews [16]. The methodological framework for scoping reviews developed by Arksey et al. was used, which comprises five stages: identifying the research question, identifying relevant studies, selecting studies, charting the data and collating, summarizing and reporting the results. Ethical approval was not necessary for this scoping review because a literature review did not require ethical approval based on the related research guidelines.

### Eligibility criteria

The research question was constructed and developed according to the PICO framework (Population, Intervention, Comparison, Outcome). To be eligible for inclusion in this study, the reports had to meet following criteria: the study focused on the AD Children with age < 16 years old; studies focused on the preventions or treatments associated with gut microbiota; randomized controlled trials (RCTs), quasi experimental or experimental studies, cohort designs; the paper was reported in English or Chinese. We excluded narrative reviews, experts’ opinions and papers published in other languages.

### Search strategy

Two authors independently searched following databases: Pubmed, CINAHL (EBSCO), MEDLINE, Cochrane library, Cochrane central register of controlled trials, China National Knowledge Infrastructure (CNKI) and Wanfang Database, China Biomedical Literature Database (CBM) for studies focused on gut microbiota and AD in children. The search time limit was from the inception of databases to January 15, 2022. Besides, we searched Google Scholar for potentially related reports. The reference lists of included papers were also screened out to identify any additional relevant papers. The following Boolean search strategy was used to search above databases (Ti: atopic dermatitis ∗ OR dermatitis ∗) AND (Ti: microbiota ∗ OR gut microbiota ∗ OR probiotics ∗ or gastrointestinal ∗) AND (Ti: children ∗ OR child ∗ or pediatric ∗) OR (Ti: prevention ∗ OR treatment ∗). The keywords used were synonyms generated by the databases used and MeSH terms.

### Data processing and extraction

Two authors independently screened the initial papers identified through the search. The title and abstract of these papers were evaluated based on the inclusion and exclusion criteria to judge their suitability for further stages of review. The retained papers following this process were rescreened by full text to assess the eligibility. A template was developed to allow extraction of the characteristics of each of the studies. This template also allowed recording of the decision made by each reviewer as to whether the paper should be included or excluded or if the decision was uncertain. The result achieved by the two reviewers was evaluated and compared. Any disagreements were resolved by further discussion and reaching consensus.

The extracted data included the name of first author, author affiliation information, year of publication, report title, study purpose, study design, sample size, the characteristics of participant, outcome indicators, results and main conclusions.

### Data analysis

We conducted thematic synthesis proposed by Thomas et al. to answer this scoping review's research question. There are three stages in the thematic synthesis: the free line-by-line coding of the findings of primary literature; the organization of these “free codes” into related areas to construct “descriptive” themes; the development of “analytical” themes. In developing the theme of this coping review, each reviewer first performed line-by-line coding and descriptive theme generation. Furthermore, two reviewers discussed and integrated the patterns that appear in those themes to generate categories as themes as interpretations beyond the original literature's content.

## Results

### Study inclusions

**Fig. 1:**
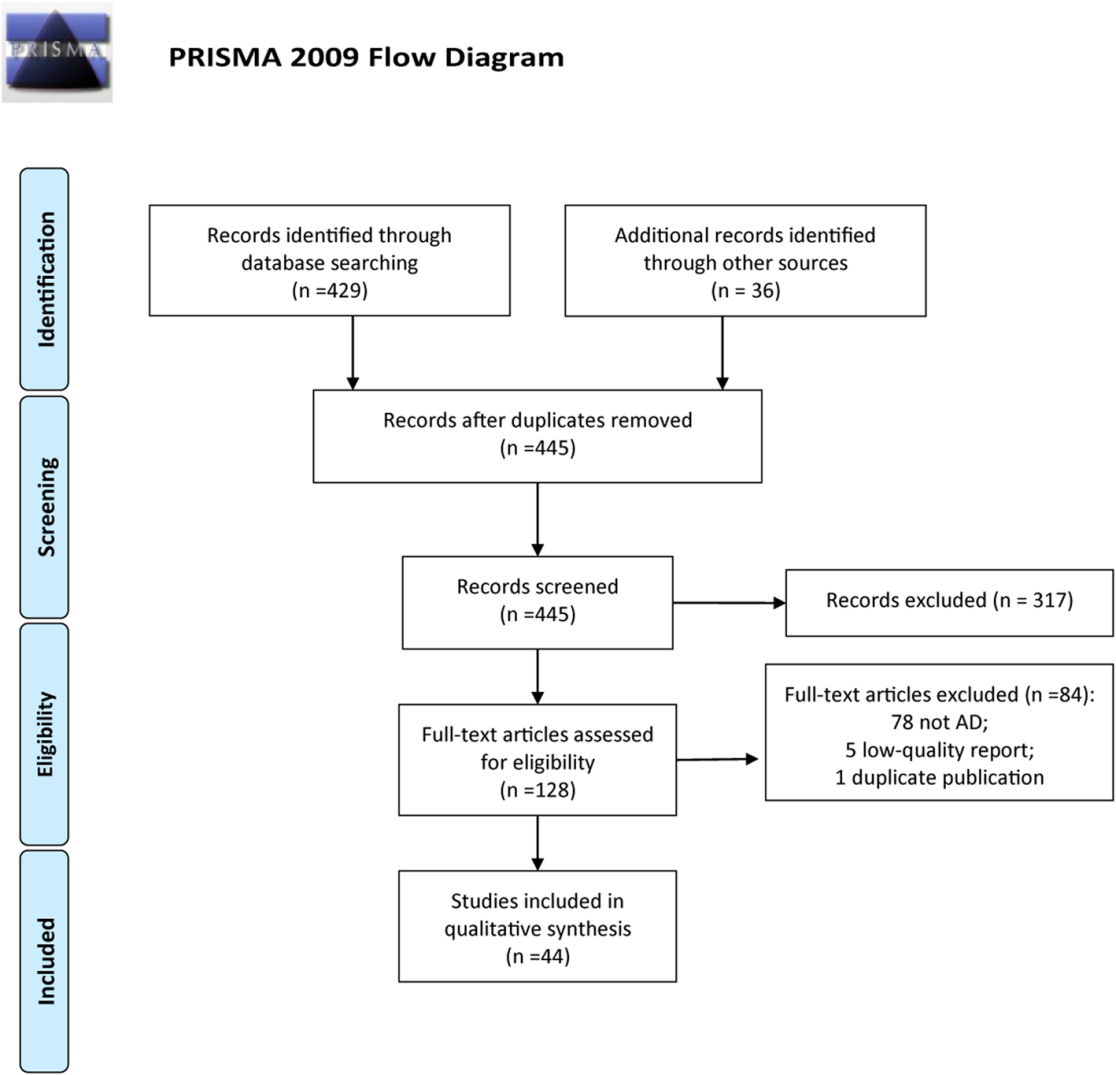
The flow diagram for literature review

The first database search yielded 465 reports for possible inclusion. Title and abstract review identified 128 articles for full-text review. After full-text review, we removed 84 additional articles for failure to meet inclusion criteria. Finally, 44 studies were included in this scoping review (Fig. 1).

### Characteristics of included literature

Of the included 44 reports, the authors were from 19 countries, the United States was the country with the most authors, followed by the United Kingdom and China. In terms of regions, Europe had the largest number of researchers, followed by Asia and south America. There were 30 observation studies and 14 intervention studies.

### Gut microbiota and AD development

The species and quantity of human microorganisms are abundant, and as symbiotic organisms, they form the human micro-ecosystem together with the host of human body. Under normal circumstances, the microbiota adheres, colonizes, and reproduces in specific parts of the human body, forming a biofilm barrier, thereby inhibiting the invasion of pathogenic bacteria and maintaining the dynamic balance between the human body and microorganisms [17, 18]. The classic “hygiene hypothesis” holds that urbanized lifestyles, excessive hygiene, and the widespread use of antibiotics can lead to imbalances in human microecology and induce or aggravate allergic diseases such as AD [19]. The skin and the gut are the two main micro-ecosystems of the human body. Some studies have found that compared with the normal population, the diversity of skin and intestinal microbiota in children with AD is reduced before the onset of the disease, which can lead to a shift in the immune response to the T helper 2 (Th2) and increase the possibility of AD onset [20, 21].

During the investigation of Swedish and Estonian children [22, 23], Estonian children had very few allergies, and their intestinal microbiota was dominated by Lactobacillus. However, the main intestinal microbiota of Swedish children was Clostridium, so there were many allergic symptoms. For non-allergic individuals, allergic infants had low levels of lactobacilli in their guts, along with a large number of aerobic bacteria. The results of in vitro and in vivo experiments [24, 25] showed that *Lactobacillus* could regulate the immune system and resist allergy. Elevated fecal caproic acid levels suggest altered gut microbial communities in infants with allergic symptoms [26, 27]. Experimental data [28] had showed that the intestinal bifidobacteria, gram-positive aerobic bacteria and enterococci were relatively few, but children with a high number of *Clostridium* and *Staphylococcus aureus* are prone to allergic symptoms.

### Probiotics and AD

Probiotics are live microorganisms that help to promote the balance of microbiota, have immune regulation, antioxidant and antibacterial effects, and have been widely used in the prevention and treatment of allergic diseases in children [29]. Th2-type inflammation is the basic feature of AD [30]. On the one hand, oral probiotics can inhibit the Th2 response and make the immune response develop in the direction of Th1 [31]. On the other hand, it can induce immune tolerance and maintain the balance of Th1/Th2, so as to achieve the effect of treating AD [32, 33]. Previous studies have mostly focused on oral probiotics, and topical and oral probiotics are the new hotspots in the research on the relationship between microbiota and AD in recent years. It has been found that oral probiotics can reduce the abnormal colonization of Staphylococcus aureus and other bacteria, restore the diversity of microbiota, help the recovery of intestinal barrier function, and alleviate the clinical symptoms of AD [34].

Epidemiological surveys [35, 36] showed that the proportions of *Clostridium, Clostridium difficile, Escherichia coli* and *Staphylococcus aureus* in the gut microbiota of AD patients increased, while the proportions of *Bifidobacterium* and *Bacteroides* decreased, suggesting the pathogenesis of AD is closely related to the disturbance of intestinal microbiota. In 2020, Jiang et al. [37] conducted a meta-analysis on the role of probiotics in the prevention and treatment of AD in children, proving that oral probiotics in pregnant women and newborns can effectively reduce the prevalence of AD in children, and children with AD take probiotics orally. It can effectively relieve its clinical symptoms and improve the quality of life. Clinical studies by Navarro-López et al. [38] has found that AD children over 1 year old responded better to oral probiotics, while the efficacy of oral probiotics in children with moderate to severe AD was better than that in children with mild AD. In addition, multi-strain preparations, especially the probiotic mixture containing *lactobacillus* and *bifidobacteria* that has a better therapeutic effect than a single strain preparation.

The RCT of Tan-Lim et al. [39] has showed that both single-strain preparations and multi-strain mixed preparations can effectively reduce the clinical symptoms of AD in children, but the multi-strain mixed preparations were more effective than single-strain preparations. Oral *Lactobacillus* acidophilus single-strain preparations or *Lactobacillus acidophilus* mixed preparations with other strains of probiotics can improve clinical symptoms in children with AD [34, 40]. The combined use of *Lactobacillus casei* and *Lactobacillus salivarius* can reduce IgE levels [41]. Prakoeswa et al. [42]. and Kim et al. [43] applied *Lactobacillus* plantarum IS-10506 and CJLP133 to treat children with AD, respectively. After 12 weeks, the symptoms of AD children in the probiotic group were significantly improved, the SCORAD value was significantly lower than that in the placebo group, and there were no obvious adverse reactions. The study of Kim et al. [43] has found that oral probiotic preparations had a more significant effect in the treatment of AD children with elevated total IgE. It is speculated that the therapeutic effect of probiotics on AD is not only related to probiotic strains, but also to the host's immune status.

Studies [44, 45] have found that probiotic supplementation by pregnant women during prenatal to postpartum lactation and postnatal fetuses can effectively reduce the risk of AD in children, and the effect of multi-strain probiotic mixtures is better than that of single-strain formulations. Wickens et al. [46] have conducted an 11-year, double-center, randomized, double-blind, placebo-controlled trial: pregnant women in the experimental group continued to take probiotics from 35 weeks of gestation to 6 months postpartum lactation, and infants took oral probiotics from birth to 2 years of age. Pregnant women and infants in the control group were all given placebo, and the children were followed up until the age of 11. The proportion of AD in the probiotic group was significantly lower than that in the placebo group. Baldassarre et al. [45]. conducted a clinical observational trial involving 66 pregnant women. The multi-strain probiotic mixture was orally administered to pregnant women from 4 weeks before delivery to 4 weeks after delivery. The secretion of sIgA in infant feces suggests that oral probiotics during pregnancy and childbirth contribute to the improvement of intestinal barrier function in the neonatal period. Intestinal microbiota disorders are closely related to the occurrence and development of AD [47]. Oral and topical probiotics can regulate the distribution of local microbiota and improve the immune response of the body [48]. It is expected to become one of the main methods for the prevention and adjuvant treatment of AD in children.

## Discussions

Previous studies have reported that the gut of the fetus is sterile, and the growth and fixation of its intestinal microbiota does not begin until about 24 h after birth. These bacteria are affected by the mode of delivery, living and feeding environment. Anaerobic microorganisms start to multiply when breastfeeding begins to stimulate the baby's intestines [49]. Bifidobacterium, Lactobacillus and Bacteroides belong to this type of bacteria, because the oligosaccharides in breast milk stimulate the baby's stomach, so the most bacteria produced are probiotics. Its main function is to help infants absorb nutrients, minerals, promote vitamin synthesis in their body, increase the function of the immune system [50, 51]. Interfering with the denaturation of enzyme activity and lowering cholesterol are the main functions of probiotics [52–54]. The reduction of bifidobacteria in the human body and the increase of other bacteria have a certain relationship with the increase of age, but the bacteria that appear in different locations have different pathogenic properties [55]. With increasing age and gradual changes in eating habits, the intestinal microbiota in the human body will also change accordingly [56, 57]. The influence of various factors changes the changes in the microbiota in the human body, and the composition of intestinal microbes will also be changed if drugs are used for treatment [58].

In terms of dermatopathology, Luo et al. [59] have treated 80 AD children with randomized, double-blind, placebo trial, and the results have showed that after probiotic treatment, their intestinal bacteria were significantly reduced. The group was tested, and the beneficial bacteria were significantly improved, and the allergy symptoms also improved. Probiotics can effectively promote the formation of endogenous barrier mechanisms in children with atopic dermatitis, reduce intestinal inflammation, and avoid allergic symptoms. IL-10 levels increased in the children after continued treatment. Previous study [60] report randomized, double-blind, placebo trial to treat 90 children with moderate and severe atopic dermatitis as experimental subjects, and have used probiotics for adjuvant treatment, and scored itching after 16 weeks. The results have showed that, the degree of itching in children who received probiotic adjuvant therapy is significantly reduced, which have indicated that probiotic adjuvant therapy had a certain effect on the sensitivity of children's skin. At present, there are not many RCTs with rigorous design that can show that probiotics can reduce the severity of AD, and its exact therapeutic effects and safety remain to be further investigated [61].

AD is a Th2-biased inflammatory disorder caused by a combination of complex factors [62]. If the intestinal mucosal immune system is not stimulated by intestinal microorganisms in the early stage of development, the natural development of the immune system will be inhibited, and Treg cells will not be mature enough to regulate certain balances such as Th1/Th2 [11, 63]. By comparing the intestinal microbiota of infants with atopic dermatitis and healthy infants, study [64] has found that the content of Clostridium in the intestine of the former is relatively high, while the content of *Bifidobacterium* and *Lactobacillus* in the intestine of healthy infants is higher. It’s been reported that indole-3-carbaldehyde, which is a tryptophan product metabolized by gut microbiota, can alleviate skin inflammation in a mouse model of atopic dermatitis by inhibiting Th2-type cytokine secretion and IgE production. Previous study [65] has pointed out that *Bifidobacterium Lactobacillus* triple viable bacteria tablets (the ingredients are *Bifidobacterium* longum, *Lactobacillus bulgaricus* and *Streptococcus thermophilus*, 4 tablets/time, 3 times a day for a total of 12 weeks) can increase the number of AD children. These attempts to improve the disease and delay the recurrence of intestinal *bifidobacteria* and lactobacilli microbiota have brought new hope for the management of chronic disease in children with AD. It is worth noting that the balance of human microbiota is easily affected by various factors such as age, immune status, and external environment, and the therapeutic effect of probiotics is closely related to the strain, timing and dose of administration [66–68]. Large-scale randomized, double-blind, placebo-controlled clinical trials are still needed to determine the effective strains, appropriate doses, and duration of treatment of probiotics for AD prevention and treatment.

Several review articles about probiotics and gut microbiota with AD were already published and should be considered. Fang et al. [28] have focused on the the potential mechanisms of probiotics on alleviating AD via upregulation of epidermal barrier and regulation of immune signaling, and the possible effective substances on AD, which provide the supports for targeting gut microbiota to AD prevention and treatment. Disamantiaji et al. [69] have included a total of 5 studies, and have concluded that probiotics supplementation in the management of eczema in children does not show a clinically relevant difference vs. standard treatment in reducing eczema severity. We focused on the relationship of gut microbiota and AD in the children population, with more studies included, we have found that probiotics may be an effective and safe treatment option for AD. Still, the current research results have great heterogeneity on the role of probiotics for AD treatment. Therefore, the results of this review must be considered with some limitations. The most significant limitation was that the language of searching databases were limited to English and Chinese, there can be many studies on the gut microbiota and AD in children reported in other language. Besides, the clinical trials with rigorous design are very few, future studies with larger sample size and rigorous design are needed to elucidate the role of gut microbiota in AD development and treatment.

## Conclusions

In conclusion, the current research evidences have showed that the gut microbiota is closely related to the occurrence and development of AD. Probiotics can help immune cells to exert anti-allergic effects on AD, including enhanced antigen degradation and pro-inflammatory immune responses. Intestinal probiotics can regulate immune cells and immune factors, inhibit the reproduction of pathogens in the intestinal tract, and enhance the intestinal epithelial barrier function. It is worth noting that the specific mechanism of action related to the occurrence and development of intestinal microbiota and AD is not yet clear. Besides, the therapeutic effect and safety of probiotics in children with AD still need to be further confirmed.

## Data Availability

All data generated or analyzed during this study are included in this published article.
